# Cross-cancer evaluation of polygenic risk scores for 16 cancer types in two large cohorts

**DOI:** 10.1038/s41467-021-21288-z

**Published:** 2021-02-12

**Authors:** Rebecca E. Graff, Taylor B. Cavazos, Khanh K. Thai, Linda Kachuri, Sara R. Rashkin, Joshua D. Hoffman, Stacey E. Alexeeff, Maruta Blatchins, Travis J. Meyers, Lancelote Leong, Caroline G. Tai, Nima C. Emami, Douglas A. Corley, Lawrence H. Kushi, Elad Ziv, Stephen K. Van Den Eeden, Eric Jorgenson, Thomas J. Hoffmann, Laurel A. Habel, John S. Witte, Lori C. Sakoda

**Affiliations:** 1 Department of Epidemiology and Biostatistics, University of California San Francisco, San Francisco, CA USA; 2 Division of Research, Kaiser Permanente Northern California, Oakland, CA USA; 3 Helen Diller Family Comprehensive Cancer Center, University of California San Francisco, San Francisco, CA USA; 4 Program in Biological and Medical Informatics, University of California San Francisco, San Francisco, CA USA; 5 Institute for Human Genetics, University of California San Francisco, San Francisco, CA USA; 6 Department of Medicine, University of California San Francisco, San Francisco, CA USA; 7 Department of Urology, University of California San Francisco, San Francisco, CA USA; 8 Department of Health System Science, Kaiser Permanente Bernard J. Tyson School of Medicine, Pasadena, CA USA

**Keywords:** Cancer epidemiology, Cancer genetics, Cancer genetics

## Abstract

Even distinct cancer types share biological hallmarks. Here, we investigate polygenic risk score (PRS)-specific pleiotropy across 16 cancers in European ancestry individuals from the Genetic Epidemiology Research on Adult Health and Aging cohort (16,012 cases, 50,552 controls) and UK Biobank (48,969 cases, 359,802 controls). Within cohorts, each PRS is evaluated in multivariable logistic regression models against all other cancer types. Results are then meta-analyzed across cohorts. Ten positive and one inverse cross-cancer associations are found after multiple testing correction. Two pairs show bidirectional associations; the melanoma PRS is positively associated with oral cavity/pharyngeal cancer and vice versa, whereas the lung cancer PRS is positively associated with oral cavity/pharyngeal cancer, and the oral cavity/pharyngeal cancer PRS is inversely associated with lung cancer. Overall, we validate known, and uncover previously unreported, patterns of pleiotropy that have the potential to inform investigations of risk prediction, shared etiology, and precision cancer prevention strategies.

## Abstract (web summary)

While genetic loci shared between cancer types have been identified, cross-cancer relationships for polygenic risk scores have not been well studied. Here, the authors have developed polygenic risk scores for 16 cancers in two large cohorts and identified positive and inverse cross-cancer associations.

## Introduction

Neoplasms are remarkably diverse in their clinical presentation, but they share biological hallmarks acquired during the transformation of normal cells into neoplastic ones^1^. Inherited genetic factors underpinning shared hallmarks could alter cancer risk in a pleiotropic manner. Several high-penetrance mutations have been shown to exhibit pleiotropy across multiple cancers; *BRCA2*, for example, a gene involved in DNA repair, has been implicated in cancers of the breast, ovary, pancreas, and prostate^2^. Genome-wide association studies (GWAS) of individual cancer types have also identified loci associated with multiple cancer types, including 5p15 (*TERT-CLPTM1L)*^3^, 6p21 (HLA complex)^4,5^, and 8q24^6^. Non-GWAS approaches have yielded further pleiotropic cancer risk variants^7–27^, and genetic correlation studies have identified cancer pairs with shared heritability^28–31^.

Polygenic risk scores (PRS) capture a different aspect of pleiotropy. By combining variants into scores that summarize genetic susceptibility, PRS typically explain a larger proportion of disease risk than single low-penetrance variants. Relative to genetic correlations, PRS offer greater specificity by selecting a refined set of disease-specific risk variants. PRS analyses therefore have the potential to enhance clinical risk assessment; given diagnosis with the first of two cancers with known shared genetic susceptibility, it could prove prudent to more aggressively consider primary prevention and screening efforts for second cancer in the pair. Beyond prediction, biological insights into underlying etiologic mechanisms may be gained by investigating the functionality of shared variants. Although PRS have been extensively investigated for individual cancers, the cross-cancer portability of PRS has been less well studied.

In this work, we comprehensively investigate pleiotropy across cancers by leveraging results from 257 published GWAS to systematically construct PRS specific to 16 cancer types. We then evaluate associations between each PRS and the risk of each cancer type in European ancestry individuals from two large independent cohorts with genome-wide array data—the Genetic Epidemiology Research on Adult Health and Aging (GERA) cohort and the UK Biobank. We also assess associations between each genetic variant contributing to a PRS and the risk of each cancer type and, among UK Biobank participants, characterize pleiotropy between each PRS and 20 cancer risk factors or biomarkers. We validate known and uncover previously unreported patterns of pleiotropy that have the potential to inform investigations of risk prediction, shared etiology, and precision cancer prevention strategies.

## Results

**Fig. 1:**
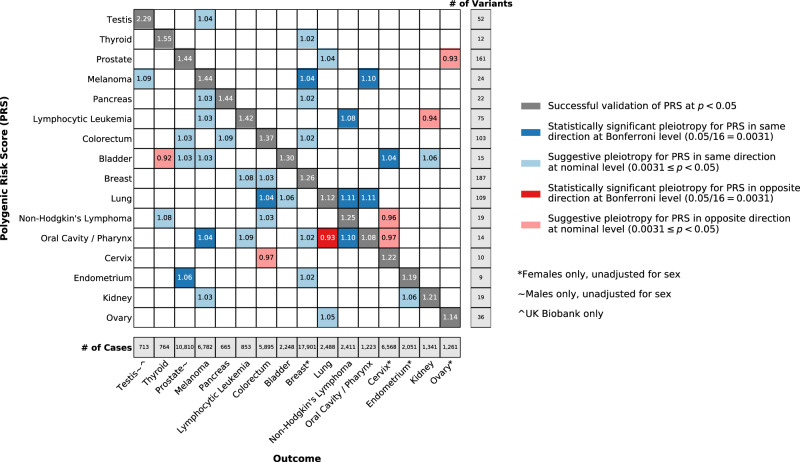
Odds ratios for at least nominally significant associations between cancer-specific polygenic risk scores (PRS) and cancer outcomes, based on meta-analyses of European ancestry participants from the Genetic Epidemiology Research on Adult Health and Aging (GERA) cohort and UK Biobank. Odds ratios were estimated from logistic regression models, *p* values were calculated from two-sided Wald tests, and statistical significance (*p* < 0.05/16 = 0.0031) was determined accounting for multiple testing. Cancers are ordered based on hierarchical clustering of the odds ratios for each PRS across cancer outcomes.

In a first step toward development of PRS for each of 16 cancer types, we abstracted 17,717 genome-wide significant associations from 257 published GWAS (Supplementary Data 1). Of the selected set of 867 risk variants independent (*r*^2^ < 0.3) within the 16 cancer types, 798 variants were independent across all cancer types (Supplementary Data 2a–p). Endometrial cancer had the fewest independent risk variants (*n* = 9), and breast cancer had the most (*n* = 187) (Fig. 1).

Participants were more commonly female than male (Supplementary Table 1). GERA participants were older than UK Biobank participants (mean age in years: cases, 69 versus 60; controls, 62 versus 57). Case counts ranged from 665 for pancreatic cancer to 17,901 for breast cancer (Fig. 1; we restricted to cancers with at least 650 cases across cohorts). Meta-analyses of non-sex-specific cancers included 410,354 controls. Female-specific meta-analyses included 219,648 controls. Meta-analyses of prostate cancer included 190,706 male controls. For testicular cancer, analyses included 169,967 male controls (UK Biobank only).

### PRS replication within cancer types

Initial analyses assessed each of the 16 PRS in relation to the cancer for which it was developed. All PRS replicated at a nominal significance level (*p* < 0.05; dark gray cells in Fig. 1) for their corresponding cancer outcomes. The largest effect sizes per standard deviation increase in the PRS were observed for testicular (odds ratio (OR) = 2.29; *p* = 6.82 × 10^−105^) and thyroid cancers (OR = 1.55; *p* = 6.38 × 10^−33^). The smallest were observed for ovarian (OR = 1.14; *p* = 2.72 × 10^−6^) and oral cavity/pharyngeal cancers (OR = 1.08; *p* = 0.007). None of these replicative associations demonstrated significant heterogeneity across cohorts (*p*_Cochran’s-Q_ < 0.05). Supplementary Data 3a–c include summary statistics from the meta-analyses, GERA, and UK Biobank, respectively.

### Cross-cancer PRS associations

Next, we evaluated each PRS in relation to each of the other 15 cancer types. Eleven associations between a PRS and cross-cancer outcome were found after correction for multiple testing (*p* < 0.05/16 = 0.0031; Fig. 1). Results remained materially unchanged correcting for the false discovery rate at *q* < 0.05 (Supplementary Fig. 1). Ten pairs showed a positive association: bladder cancer PRS with cervical cancer (OR = 1.04; *p* = 9.04 × 10^−4^); endometrial cancer PRS with prostate cancer (OR = 1.06; *p* = 5.34 × 10^−9^); lung cancer PRS with non-Hodgkin’s lymphoma (NHL; OR = 1.11; *p* = 5.57 × 10^−7^), colorectal cancer (OR = 1.04; *p* = 1.22 × 10^−3^), and oral cavity/pharyngeal cancer (OR = 1.11; *p* = 1.06 × 10^−4^); lymphocytic leukemia PRS with NHL (OR = 1.08; *p* = 1.48 × 10^−4^); melanoma PRS with breast (OR = 1.04; *p* = 6.33 × 10^−7^) and oral cavity/pharyngeal cancers (OR = 1.10; *p* = 7.84 × 10^−4^); and oral cavity/pharyngeal cancer PRS with melanoma (OR = 1.04; *p* = 2.04 × 10^−3^) and NHL (OR = 1.10; *p* = 2.67 × 10^−6^). The oral cavity/pharyngeal cancer PRS was inversely associated with lung cancer (OR = 0.93*; p* = 6.25 × 10^−4^). Only the melanoma PRS-breast cancer association demonstrated heterogeneity (*I*^*2*^ = 0.79; *p*_Cochran’s-Q_ = 0.029). Thirty additional associations (24 positive, six inverse) were nominally significant (*p* < 0.05).

In sensitivity analyses removing variants from the exposure PRS in linkage disequilibrium (LD) with variants known to be associated with the outcome cancer type (Supplementary Data 4 and Supplementary Table 2), 8 out of the 11 significant associations remained materially unchanged (Supplementary Table 3). Three relationships, however, were seemingly driven by variants in LD: endometrial cancer PRS with prostate cancer (OR = 1.00; *p* = 0.96); oral cavity/pharyngeal cancer PRS with melanoma (OR = 1.02; *p* = 0.075); and oral cavity/pharyngeal cancer PRS with lung cancer (OR = 0.98; *p* = 0.25). The original oral cavity/pharyngeal cancer PRS was significantly associated with lung adenocarcinoma (1006 cases; OR = 0.89; *p* = 1.13 × 10^−4^) but not squamous cell carcinoma (488 cases; OR = 0.94; *p* = 0.20). When restricting the lymphocytic leukemia PRS to variants discovered in GWAS of chronic lymphocytic leukemia (CLL), the relationship with NHL became more pronounced (OR = 1.10; *p* = 1.97 × 10^−6^). When excluding 20 lung cancer risk variants associated with tobacco use^32^, associations of the lung cancer PRS with non-Hodgkin’s lymphoma (OR = 1.11; *p* = 6.26 × 10^−8^), colorectal cancer (OR = 1.05; *p* = 6.11 × 10^−5^), and oral cavity/pharyngeal cancer (OR = 1.12; *p* = 3.95 × 10^−5^) remained unchanged. No other PRS implicated in cross-cancer associations included tobacco-associated variants.

### Cross-cancer risk variant associations

**Fig. 2:**
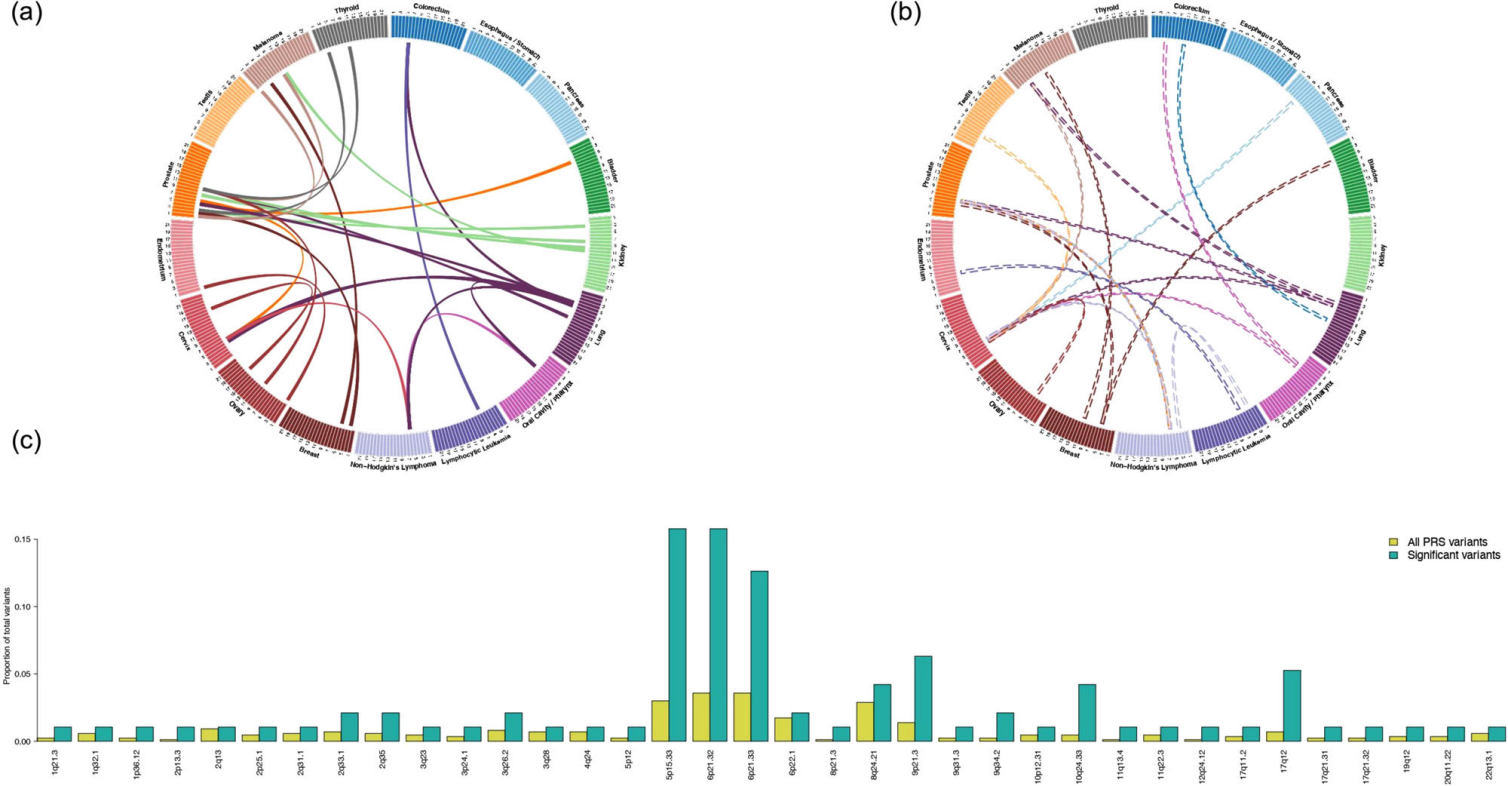
Pleiotropic risk variants from the 16 cancer-specific polygenic risk scores (PRS). **a** Circos plot describing each previously unreported positive association between a known risk variant for one cancer type and another cancer phenotype. **b** Circos plot describing each previously unreported inverse association between a known risk variant for one cancer type and another cancer phenotype. Each line in **a** and **b** represents a significant association between a risk variant for the cancer from which the line originates (denoted by line color) and the cancer type to which the line connects. Odds ratios were estimated from logistic regression models, *p* values were calculated from two-sided Wald tests, and statistical significance (*p* < 0.05/798 = 6.3 × 10^−5^) was determined correcting for the number of effective independent tests. Cancers are organized by organ site. **c** Region enrichment for 141 significant previously unreported and known associations compared to all PRS variants.

We further assessed associations between each variant contributing to a PRS and all cancer types (Supplementary Data 5a–p). In total, 141 cross-cancer associations were detected at a threshold corrected for the number of effective independent tests (*p* < 0.05/798 = 6.3 × 10^−5^; Supplementary Data 6; includes 18 duplicate associations in which the same variant originated from multiple PRS). They included associations for 55 variants in LD with previously identified risk variants for the outcome cancer. Among the remaining 86 associations, 60 were previously unreported, in that prior literature had not reported the variant (or variants with *r*^2^ > 0.3 in the 1000 Genomes EUR superpopulation reference panel) to be associated with the outcome cancer at *p* < 1 × 10^−6^ (Fig. 2a and 2b; includes five duplicate associations originating from multiple PRS). The cancer types with the largest number of such risk variants were prostate (*n* = 15), NHL (*n* = 14; includes one variant originating from multiple PRS), and cervix (*n* = 12).

Several genomic regions were overrepresented among pleiotropic variants compared with all PRS variants (Fig. 2c). Across the 141 cross-cancer associations, pleiotropic variants were most commonly found in *TERT-CLPTM1L* (16% versus 3.0%) and HLA (6p21.32: 16% versus 3.6%; 6p21.33: 13% versus 3.6%). Additional regions enriched for pleiotropy included 9q34.2 (2.1% versus 0.23%), 10q24.33 (4.2% versus 0.46%), 12q24.12 (1.1% versus 0.11%), and 17q12 (5.3% versus 0.69%). These regions remained enriched following normalization by region size (Supplementary Fig. 2).

### Cancer PRS and cancer risk factors or biomarkers

**Fig. 3:**
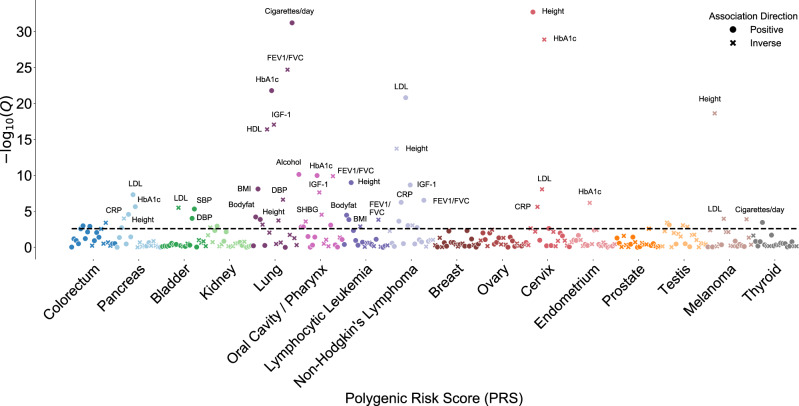
Associations between each cancer-specific polygenic risk score (PRS) and 20 cancer risk factors and related serum biomarkers. All associations were estimated in cancer-free controls in the UK Biobank. Circles denote positive associations between the PRS and the secondary phenotype; crosses denote an inverse direction of the association. Odds ratios (ever/never smoking status) were estimated from logistic regression models, and *p* values were calculated from two-sided Wald tests. Risk ratios (remaining phenotypes) were estimated from linear regression models, and *p* values were calculated from two-sided *t* tests. The dashed line indicates the significance threshold corrected for multiple testing (*p* < 0.05/20 = 0.0025).

Upon evaluating relationships between each cancer PRS and 20 cancer risk factors or biomarkers in the UK Biobank, we identified 62 statistically significant associations (*p* < 0.05/20 = 0.0025; Fig. 3, Supplementary Data 7). The lung cancer PRS was associated with the most (12) phenotypes. Positively associated phenotypes included cigarettes per day in smokers (*p* = 6.06 × 10^−32^), pulmonary obstruction (decreasing forced expiratory volume in 1 s [FEV_1_]/ forced vital capacity [FVC]; *p* = 1.97 × 10^−25^), glycated hemoglobin (HbA1c; *p* = 1.59 × 10^−22^), height (*p* = 1.30 × 10^−4^), and multiple metrics of adiposity (e.g., body mass index (BMI): *p* = 7.63 × 10^−9^). The lung cancer PRS was associated with lower levels of insulin-like growth factor-1 (IGF-1) (*p* = 8.58 × 10^−18^) and high-density lipoprotein (HDL) cholesterol (*p* = 3.94 × 10^−17^). The NHL and oral cavity/pharyngeal cancer PRS were each associated with nine secondary phenotypes. Among the associations for the former were increasing levels of low-density lipoprotein (LDL) cholesterol (*p* = 1.53 × 10^−21^), IGF-1 (*p* = 2.13 × 10^−9^), and C-reactive protein (CRP; *p* = 5.50 × 10^−7^). The latter was associated with increasing alcohol intake (*p* = 7.28 × 10^−11^) and pulmonary obstruction (*p* = 1.26 × 10^−10^). PRS for breast, prostate, and ovarian cancers were not clearly associated with any secondary phenotypes. Among the secondary phenotypes, height showed the most cancer PRS associations (*n* = 8; 4 positive, 4 inverse), followed by HbA1c (*n* = 7; 5 positive, 2 inverse), and BMI (4 positive, 2 inverse) and LDL (2 positive, 4 inverse) (*n* = 6 each).

## Discussion

In this comprehensive study of PRS-specific cancer pleiotropy, we constructed 16 PRS based on systematic review of the cancer GWAS literature. Analyses identified 11 statistically significant cross-cancer PRS associations, as well as previously unreported cancer associations with 55 unique risk variants in known susceptibility regions. We further identified 62 cancer PRS associations with selected non-cancer phenotypes.

Of all PRS evaluated, the oral cavity/pharyngeal and lung cancer PRS were most commonly implicated in associations with cross-cancer and non-cancer phenotypes. These results support existing evidence of cancer pleiotropy, given that the PRS for both cancers included variants in two well-known pleiotropic cancer regions—*TERT*-*CLPTM1L*^3^ and HLA^4,5^. Notwithstanding shared susceptibility regions, the relationship between oral cavity/pharyngeal and lung cancers was inconsistent. In one direction, the oral cavity/pharyngeal cancer PRS was inversely associated with lung cancer. The negative pleiotropy was at least partly attributable to two oral cavity/pharyngeal PRS variants (rs467095 and rs10462706; Supplementary Data 4 and Supplementary Table 2), both expression quantitative trait loci for *TERT* and *CLPTM1L*, which were inversely associated with lung cancer risk and in LD (*r*^2^ = 0.96 and 0.66, respectively) with variants in the lung cancer PRS. The oral cavity/pharyngeal cancer PRS was also associated with increasing alcohol intake, an established risk factor for such cancers^33^. In contrast, the relationship between alcohol intake and lung cancer remains controversial, with the possibility of an inverse or J-shaped relationship^34,35^. In the other direction, the lung cancer PRS was positively associated with oral cavity/pharyngeal cancer risk. The positive pleiotropy may be partially explained by the association between both cancers and pulmonary obstruction (i.e., decreasing FEV_1_/FVC), as well as higher HbA1c and lower IGF-1 levels, both of which indicate insulin resistance. The association remained largely unchanged after excluding smoking-associated variants, suggesting that tobacco use unlikely explains it.

Oral cavity/pharyngeal cancer also showed a bidirectional, positive relationship with melanoma, even though the two PRS share only one pair of variants in LD in *TERT*-*CLPTM1L* (Supplementary Data 4 and Supplementary Table 2). PRS for both cancer types were inversely associated with height, which is somewhat surprising since increasing height has been strongly associated with melanoma risk^36^.

The lung cancer PRS was positively associated with colorectal cancer and NHL. The former association did not appear to be driven by variants in LD; only two out of 109 lung cancer risk variants (rs2853677 and rs1333040) are in high LD (*r*^2^ = 0.62 and 0.49, respectively) with colorectal cancer risk variants (rs2735940 and rs1537372, respectively), and neither was strongly associated with colorectal cancer risk in our data. Given that the lung cancer PRS was associated with increasing BMI, body fat, and cigarettes per day, its association with colorectal cancer risk coheres with known risk factors. As five of the lung cancer variants in HLA are in LD with NHL risk variants, LD structure likely played a larger role in the latter association, though it was only slightly weaker after excluding all SNPs in LD. One possible shared mechanism is insulin resistance, as both the lung cancer and NHL PRS were associated with increasing HbA1c levels. We also identified a previously unreported association between a lung cancer risk variant and NHL; rs652888 (6p21.33 in *EHMT2*) has been linked to several autoimmune and infectious diseases^37,38^, as well as infection with Epstein-Barr virus^39^, a known NHL risk factor^40^. Although we did not find an association between the NHL PRS and lung cancer risk, the NHL PRS included only 19 SNPs (relative to 109 variants in the lung cancer PRS).

Among the remaining significant cross-cancer PRS associations, two included cancers with PRS variants that were completely independent at the *r*^2^ = 0.3 threshold: the bladder cancer PRS with cervical cancer and the oral cavity/pharyngeal cancer PRS with NHL. Cervical cancer and NHL were among the cancers with the most previously unreported risk variants. Although none of the 15 bladder cancer variants are in LD with known genome-wide significant risk variants for cervical cancer, one *CLPTM1L* variant (rs401681-C) was associated with increased cervical cancer risk at a genome-wide significance level in our study, confirming a suggestive association signal reported previously^22^. Similarly, two oral cavity/pharyngeal cancer variants in HLA (rs9271378 and rs3135006), a region that has previously been implicated in NHL^41^, were strongly associated with NHL risk in our analyses.

Increasing NHL risk was also associated with the overall lymphocytic leukemia and CLL PRS. Out of 64 lymphocytic leukemia risk variants, only one (rs4987855) is in LD (*r*^2^ = 0.95) with an NHL risk variant (rs17749561). Our results align with those from Sampson et al.^30^, which showed an association between a PRS for CLL and the risk of diffuse large B-cell lymphoma, the most common NHL subtype. Both CLL and NHL arise from B-cells, and recent classifications account for their similar origin^42^.

The association between the endometrial cancer PRS and prostate cancer risk, which we attributed to one endometrial cancer variant (rs11263763) in LD with a prostate cancer risk variant (rs4430796), also validated results from Sampson et al.^30^. The remaining cross-cancer association from our study—between the melanoma PRS and breast cancer—was not evaluated in theirs. Their study did, however, identify two associations that our analyses did not validate: (1) a lung cancer PRS and bladder cancer risk, and (2) an endometrial cancer PRS and testicular cancer risk. Given differences in study design and the many additional SNPs that have been discovered since 2015, it is not especially surprising that some results are distinct.

Overall, the results of our cross-cancer PRS analyses demonstrated unique patterns of pleiotropy relative to studies focused on array-based shared heritability or genetic correlations^28–31^. In GERA and the UK Biobank, our group previously found suggestive evidence of positive correlations between bladder and breast cancers, melanoma and testicular cancer, and prostate and thyroid cancers and negative correlations between endometrial and testicular cancers, lung cancer and melanoma, and NHL and prostate cancer^31^. These pairs did not surface in our cross-cancer PRS analyses in these same cohorts, except suggestive evidence of bidirectional positive associations between melanoma and testicular cancer. By capturing a broad polygenic signal, genetic correlations offer an overall sense of genetic sharing between two cancers. In contrast, PRS capture only the strongest risk variants for a given phenotype (which may in part be informed by the number of cases examined in contributing discovery GWAS). PRS thus enable more targeted analyses of cross-cancer susceptibility loci and have more clinical potential for individual risk assessment.

Our analyses identifying genomic regions overrepresented among pleiotropic variants solidify results that have been synthesized from GWAS of individual cancers. In addition to *TERT-CLPTM1L* and HLA, 9q34.2, 10q24.33, 12q24.12, and 17q12 have been implicated in susceptibility for multiple cancer types. Variants in the breast^43^ and pancreatic cancer^44^ susceptibility locus 9q34.2 influence estrogen receptor signaling and insulin resistance, and were recently associated with protein biomarkers affecting carcinogenesis^45^. The 10q24.33 region containing *OBFC1*, a known telomere maintenance gene, has been implicated in lymphocytic leukemia, melanoma, and kidney, ovarian, and thyroid cancers^46–51^. A previous cross-cancer analysis linked 12q24.12 to both colorectal and endometrial cancer risk^52^. This locus includes *SH2B3*, a gene involved in regulating signaling pathways related to hematopoiesis, inflammation, and cell migration. The 17q12 locus includes *HNF1B*, which has been extensively characterized with respect to hormonally driven cancers^53^.

The non-cancer phenotypes that most frequently surfaced in associations with cancer PRS offer additional mechanistic insights. For example, the lymphocytic leukemia, NHL, and kidney, lung, oral cavity/pharyngeal, and pancreatic cancer PRS were associated with at least one anthropometric trait and showed directionally consistent associations with HbA1c and IGF-1 levels. Obesity-induced chronic inflammation and oxidative stress create a milieu conducive to malignant transformation^54^. Furthermore, the metabolic reprogramming necessary to meet the increased energy requirements of proliferating malignant cells is a known hallmark of cancer^1^. There is also complex interplay between genetic determinants of adiposity and smoking behaviors^55^. Taken together, the findings further implicate obesity-related metabolic dysregulation in susceptibility to multiple types of cancer. The PRS for some types of cancer, however, were not associated with any secondary phenotypes. In the case of the prostate cancer PRS, lacking associations could be a reflection of the very few established risk factors for disease. For the breast and ovarian cancer PRS, it is perhaps more likely that the distinct risk variants reflect heterogeneous etiologies.

Among the limitations of our study was the inclusion of exclusively European ancestry individuals; results may not be generalizable to diverse populations. We were also limited by modest numbers for some cancers. We favored their inclusion in an effort to evaluate more cancer types than previous investigations. Because they tend to be rarer cancers across study populations, the GWAS in which their risk variants have been identified have had smaller sample sizes than those for more common cancers. As a result, fewer risk variants have been discovered and our associated PRS may be less precise. Nonetheless, the thyroid cancer PRS, which included only 12 variants, was among the most strongly associated with the cancer for which it was developed. We note that our analyses included prevalent and incident cases. However, results from a posteriori cross-cancer PRS analyses restricted to incident and, separately, prevalent cases mirrored those from the primary analyses (Supplementary Data 8). Our findings are thus unlikely to be driven by associations with survival rather than risk. We also note that our PRS were comprised of exclusively genome-wide significant variants. Although a less-stringent threshold for inclusion might have yielded more signal, it would not have been based on convincing a priori evidence. Finally, although all PRS replicated for their target cancers, some individual risk variants did not. Nevertheless, 93% had effect estimates with consistent directionality relative to the published literature.

Among the strengths of our study was the use of two large cohorts with abundant individual-level genetic and phenotypic data, independent of those from which risk variants were identified in prior cancer GWAS (except the use of GERA data in Hoffmann et al.^56^ and limited use of UK Biobank data in Huyghe et al.^57^; see Methods). We also comprehensively reviewed the contemporary literature to identify genome-wide significant risk variants for 16 cancer types. Evaluating risk variants identified for one cancer with respect to risk for others enabled the discovery of susceptibility loci that would not otherwise meet the strict criteria for genome-wide significance. By additionally evaluating associations with cancer risk factors, we generated insights into pathways that may be influenced by genetic variants implicated in cancer.

Our work expands the repertoire of genetic susceptibility variants for multiple cancers, which can guide future investigations of their biological and clinical relevance. Although the precise biological mechanisms underpinning the associations remain ambiguous, our findings may still be leveraged toward a more integrated model of cancer risk prediction that considers cross-phenotype effects in addition to cancer-specific risk factors. An approach that incorporates genetic susceptibility profiles may have the greatest potential to aid in risk prediction for cancers with few modifiable risk factors. Combined with future research that investigates pleiotropy in cancer subgroups (e.g., by smoking status or histology) and clinical applications of PRS, our results may inform new strategies toward reducing the burden of cancer.

## Methods

### Study populations

GERA is a prospective cohort of 102,979 adults drawn from >400,000 Kaiser Permanente Northern California (KPNC) health plan members who participated in the Research Program on Genes, Environment and Health. Participants answered a baseline survey regarding lifestyle and medical history, provided a saliva specimen between 2008 and 2011, and were successfully genotyped^58,59^. Following quality control (QC; described below), the GERA analytic population included 16,012 cases and 50,552 controls.

The UK Biobank is a population-based prospective cohort of 502,611 individuals from the United Kingdom, ages 40–69 at recruitment between 2006 and 2010^60^. Participants were evaluated at baseline visits during which assessment center staff introduced a touch-screen questionnaire, conducted a brief interview, gathered physical measurements, and collected biological samples. Following QC, the UK Biobank analytic population included 48,969 cases and 359,802 controls.

This study was approved by the KPNC and University of California Institutional Review Boards and the KP Research Bank and UK Biobank data access committees.

### Phenotyping

GERA cancer cases were identified using the KPNC Cancer Registry. Following Surveillance, Epidemiology, and End Results Program (SEER) standards, the KPNC Cancer Registry contains data on all primary cancers (i.e., diagnoses that are not secondary metastases of other cancer sites; excluding non-melanoma skin cancer) diagnosed or treated at any KPNC facility since 1988. In this study, we captured all diagnoses recorded through June 2016. Cancer cases in the UK Biobank were identified via linkage to various national cancer registries^60^. Diagnoses go as far back as the early 1970s, and the latest cancer diagnosis in our data from the UK Biobank occurred in August 2015.

In both cohorts, individuals with at least one recorded prevalent or incident diagnosis of a borderline, in situ, or malignant primary cancer were defined as cases. To align with GERA, we converted all UK Biobank diagnoses described by International Classification of Diseases (ICD)-9 or ICD-10 codes into ICD-O-3 codes. We then classified cancers in both cohorts by organ site according to the SEER site recode paradigm^61^. Because second and subsequent cancers could have been miscoded metastases of first cancer or a direct result of prior cancer treatment, we evaluated only the first primary cancer diagnosed for each individual. The analyses did, however, include 23 GERA participants and 64 UK Biobank participants who had two primary cancers diagnosed on the same date. To ensure sufficient statistical power, we grouped all oral cavity and pharyngeal cancers into single site codes. Overall, our analyses included the 16 most common site codes (>650 cases; excluding non-melanoma skin cancer) across both cohorts. Data on testicular cancer cases were obtained from the UK Biobank only owing to the small number of cases in GERA.

Controls were restricted to individuals who had no record of cancer in any of the relevant registries, who did not self-report a prior history of cancer (other than non-melanoma skin cancer) by survey, and, if deceased, who did not have cancer listed as a cause of death. For analyses of sex-specific cancer outcomes (breast, cervix, endometrium, ovary, prostate, and testis), controls were restricted to individuals of the relevant sex.

We examined PRS associations with anthropometric traits, physical measures, self-reported health-related behaviors, and serum biomarkers in the UK Biobank, where relevant data were readily available. Physical assessments yielded measures of height (Field ID: 50.0), BMI (Field ID: 21001.0), waist to hip ratio (Field ID: 48.0 divided by Field ID: 49.0), diastolic blood pressure (Field ID: 4079.0), and systolic blood pressure (Field ID: 4080.0). Body fat percentage (Field ID: 23104.0) was quantified with whole-body bio-impedance measures using the Tanita BC418MA body composition analyzer. Self-reported data on cigarette smoking and alcohol consumption were used to derive variables for smoking status (ever/never), cigarettes per day, and weekly alcohol intake (grams). We additionally evaluated eight serum biomarkers, as measured according to protocols that have been previously described^62^: CRP (mg/L), HDL cholesterol (mmol/L), LDL cholesterol (mmol/L), HbA1c (mmol/mol), IGF-1 (nmol/L), sex hormone-binding globulin (nmol/L), testosterone (nmol/L) in men, and testosterone (nmol/L) in women. All biomarker analyses were restricted to samples from the first aliquot, as these samples were least affected by unintended sample dilution issues^62^. We excluded values outside the bioanalyzer reportable range, as well as measures that required additional analytic correction due to sample handling or processing issues. CRP, HbA1c, and IGF-1 were log-transformed to achieve a normal distribution.

Phenotyping was completed using SAS v9.4 (https://support.sas.com/software/94/) and R v3.2.2 or v3.3.3 (http://www.r-project.org/).

### Genotyping and imputation

For GERA, genotyping was performed using one of four Affymetrix Axiom arrays (Affymetrix, Santa Clara, CA, USA) optimized for individuals of African, East Asian, European, and Latino race/ethnicity. Details about the array design, estimated genome-wide coverage, and QC procedures have been published previously^59,63,64^. Variants that were not directly genotyped (or excluded by QC procedures) were imputed to generate genotypic probability estimates. After pre-phasing genotypes with SHAPE-IT v2.5^65^, IMPUTE2 v2.3.1 was used to impute variants relative to the cosmopolitan reference panel from the 1000 Genomes Project (phase I integrated release; http://1000genomes.org/)^66^. Ancestry principal components (PCs) were computed using Eigenstrat v4.2^58,67^.

For the UK Biobank, genotyping was conducted for 436,839 individuals with the UK Biobank Axiom array and for 49,747 individuals with the UK BiLEVE array^60^. The former is an updated version of the latter, such that the two arrays share over 95% of their marker content. UK Biobank investigators undertook a rigorous QC protocol^60^. Imputation was performed primarily based on the Haplotype Reference Consortium reference panel, and the merged UK10K and 1000 Genomes Project (phase 3) reference panels were used for secondary data^60^. Ancestry PCs were computed using fastPCA^68^ based on a set of 407,219 unrelated samples and 147,604 genetic markers^60^.

### Quality control

Additional QC procedures included restricting to self-reported European ancestry individuals with matching self-reported and genetic sex. To further minimize population stratification, we excluded individuals for whom either of the first two ancestry PCs fell >5 standard deviations outside of the mean. We also removed samples with call rates <97%, heterozygosity >5 standard deviations from the mean, and/or first-degree relatives in the datasets.

QC was completed using a combination of R v3.2.2 or v3.3.3 (http://www.r-project.org/), PLINK v1.9 (https://www.cog-genomics.org/plink2), and KING v2.0 (http://people.virginia.edu/~wc9c/KING/).

### Variant selection for PRS

PRS were constructed based on variants associated with each cancer type in existing published GWAS. To identify relevant GWAS, we began by searching the National Human Genome Research Institute-European Bioinformatics Institute Catalog of published GWAS^69^. For every GWAS of cancer of interest (or one of its sub-phenotypes; e.g., poorly differentiated prostate cancer) that discovered at least one genome-wide significant (*p* ≤ 5 × 10^−8^) risk variant, we reviewed both the original primary manuscript and supplementary materials. We then identified additional relevant GWAS by (1) reviewing the reference section of each article and (2) searching PubMed to find other studies in which each article had been cited (Supplementary Data 1). Only two out of 257 studies identified included data that overlapped with ours. GERA was used in Hoffmann et al.^56^, which contributed effect estimates for four out of 161 variants in our prostate cancer PRS. UK Biobank data accounted for 21% of the Huyghe et al.^57^ study population and was only used in the second stage of their colorectal cancer GWAS.

After abstracting genome-wide significant variants from all studies published by 30 June 2018, we reduced the file to include one log-additive association per combination of variant identifier, phenotype/sub-phenotype, and ancestry group (Supplementary Fig. 3). For associations reported in more than one study of the same ancestry, we selected the one with a known risk allele and effect estimate with the smallest *p* value.

We retained only autosomal variants identified in populations of at least 70% European ancestry. We then excluded 2979 associations for which the source literature did not report an effect estimate and/or for which an effect allele could not be determined. For the remaining 13,793 associations, we assessed variant availability in both the GERA and UK Biobank genotypic data using QCTOOL v2 (https://www.well.ox.ac.uk/~gav/qctool_v2/) and VCFtools (http://vcftools.sourceforge.net/). For lead variants that could not be identified by variant identifier or position, we used LDlink^70^ and HaploReg^71^ to identify proxy variants with *r*^2^ ≥ 0.8. From original or proxy variants available in GERA and UK Biobank, we excluded any not in a 1000 Genomes reference population or with minor allele frequencies (MAF) that differed by >0.10, and we further restricted to biallelic risk variants with MAF ≥ 0.01. In the last step prior to LD pruning, we excluded A/T and C/G variants with MAF ≥ 0.45—due to strand flips, the appropriate effect alleles in our data could not be determined.

We used PriorityPruner^72^ and LDlink^70^ to select a set of independent risk variants with LD < 0.3 for each cancer type. (We did not require that risk variants across cancer types be independent.) The process preferentially selected variants with the smallest *p* values and highest imputation scores associated with the broadest phenotype (e.g., overall prostate cancer over poorly differentiated prostate cancer).

### Statistical analysis

For each cancer type, we calculated the PRS based on additive dosages of the individual risk variants: ∑(no. risk alleles*logOR from the literature) for *i* = 1 to *n* risk alleles. Each PRS was then standardized based on its mean and standard deviation, and evaluated in multivariable logistic regression models with respect to the cancer for which it was developed and each of the other cancer types. ORs were estimated per standard deviation increase in the PRS. Models were adjusted for age at specimen collection, first 10 ancestry PCs, sex (except models for sex-specific cancers), reagent kit used for genotyping (Axiom v1 or v2; GERA only), and genotyping array (UK Biobank only). After conducting analyses by cohort, we combined results across cohorts using fixed effects meta-analyses. Heterogeneity was assessed based on *I*^2^ and Cochran’s *Q*.

For cross-cancer relationships that were statistically significant upon meta-analysis, we conducted sensitivity analyses in which we removed variants from the exposure PRS that are in LD with variants known to be associated with the outcome cancer type. To further aid in interpreting observed associations, we also conducted sensitivity analyses examining a lymphocytic leukemia PRS that was restricted to variants discovered in GWAS of CLL specifically and PRS for each cancer that excluded smoking-associated variants. The latter selected variants for exclusion based on genome-wide significant associations in recently published meta-analyses of current versus former smoking, ever versus never smoking, cigarettes per day, and age started smoking^32^. Seventy-eight variants were unavailable from the summary statistics, but none were genome-wide significantly associated with tobacco-related phenotypes in the UK Biobank. In addition, we evaluated each PRS in relation to lung adenocarcinoma and squamous cell carcinoma separately.

For variants contributing to any of the 16 PRS, we estimated their associated risk with each cancer type using logistic regression adjusted for the aforementioned covariables. Variants were modeled individually on a log-additive scale. Results from both cohorts were meta-analyzed. We then visualized the genomic regions that were overrepresented among pleiotropic variants relative to all PRS variants.

In secondary analyses, we used the UK Biobank to explore associations between each PRS and 20 cancer risk factors or serum biomarkers. Logistic (ever/never smoking status) or linear (remaining phenotypes) regression models were restricted to cancer-free controls and adjusted for the covariables noted above, as well as cigarette pack-years (FEV_1_/FVC), assay date (serum biomarkers), and use of medications to lower cholesterol (HDL and LDL), control blood pressure (systolic and diastolic blood pressure), and regulate insulin (HbA1c).

All statistical analyses were performed using R v3.2.2 or v3.3.3 (http://www.r-project.org/).

### Reporting summary

Further information on research design is available in the Nature Research Reporting Summary linked to this article.

## Supplementary information

Supplementary Information

Peer Review

Reporting Summary

Description of Additional Supplementary Files

Supplementary Data 1-8

## Data Availability

All results from this study are available from the article or Supplementary Materials. GERA data are available via the application with a local collaborator at https://researchbank.kaiserpermanente.org/our-research/for-researchers/. UK Biobank data are publicly available from https://www.ukbiobank.ac.uk.
