# Supplementary material for: Cross-cancer evaluation of polygenic risk scores for 16 cancer types in two large cohorts
Source: Nat Commun. 2021 Feb 12;12:970. doi: 10.1038/s41467-021-21288-z (PMC7880989; doi:10.1038/s41467-021-21288-z)

Supplementary Table 1. Demographic profile of study participants in GERA and the UK Biobank

	GERA		UK Biobank	
	**Cases**	**Controls**	**Cases**	**Controls**
Total, n	16,012	50,552	48,969	359,802
Sex, n (%)				
Female	8,709 (54%)	29,813 (59%)	30,385 (62%)	189,835 (53%)
Male	7,303 (46%)	20,739 (41%)	18,584 (38%)	169,967 (47%)
Mean Age at Specimen Collection, years (SD)	69 (11)	62 (13)	60 (7.2)	57 (8.0)
Reagent Kit, n (%)				
Axiom v1	15,271 (95%)	48,445 (96%)	N/A	N/A
Axiom v2	741 (5%)	2,107 (4%)	N/A	N/A
Array, n (%)				
UK Biobank Axiom	N/A	N/A	43,363 (89%)	320,883 (89%)
UK BiLEVE	N/A	N/A	5,606 (11%)	38,919 (11%)

Supplementary Table 2. Linkage disequilibrium between variants in the polygenic risk scores for significantly associated cross-cancer pairs

		Bonferroni Significant Variants		Nominally Significant Variants		Nonsignificant Variants	
**PRS**	**Outcome**	**# in LD[a]**	**# in Low LD[b]**	**# in LD[a]**	**# in Low LD[b]**	**# in LD[a]**	**# in Low LD[b]**
Bladder	Cervix	0	1	0	5	0	9
Endometrium	Prostate	1	0	0	0	0	8
Lung	Colorectum	0	3	0	31	2	73
Lung	Non-Hodgkin's Lymphoma	2	10	2	21	1	73
Lung	Oral Cavity / Pharynx	0	2	3	24	3	77
Lymphocytic Leukemia	Non-Hodgkin's Lymphoma	0	3	1	7	0	64
Melanoma	Breast	1	0	1	4	0	18
Melanoma	Oral Cavity / Pharynx	0	0	0	3	1	20
Oral Cavity / Pharynx	Lung	1	0	2	0	1	10
Oral Cavity / Pharynx	Melanoma	1	0	0	5	0	8
Oral Cavity / Pharynx	Non-Hodgkin's Lymphoma	0	2	0	1	0	11

Abbreviations: LD - Linkage Disequilibrium

[a] $r^2 \geq 0.3$

[b] $r^2 < 0.3$

Supplementary Table 3. Results from sensitivity meta-analyses of significant cross-cancer pairs (Figure 1), excluding variants from the exposure polygenic risk score in linkage disequilibrium with variants known to be associated with the outcome cancer type. Odds ratios were estimated from logistic regression models, *p*-values were calculated from two-sided Wald tests, and statistical significance ($p<0.05/16=0.0031$) was determined accounting for multiple testing.

Polygenic Risk Score	# of Variants	Outcome	# Cases	# Controls	Odds Ratio	Lower Bound	Upper Bound	*p*-value	I^2	Q *p*-value
Bladder	15	Cervix	6568	219648	1.04	1.02	1.07	9.04E-04	0.00	9.98E-01
Endometrium	8	Prostate	10810	190706	1.00	0.98	1.02	9.62E-01	0.39	2.00E-01
Lung	107	Colorectum	5895	410354	1.04	1.02	1.07	1.27E-03	0.00	8.27E-01
Lung	104	Non-Hodgkin's Lymphoma	2411	410354	1.10	1.06	1.14	1.72E-06	0.00	6.55E-01
Lung	103	Oral Cavity / Pharynx	1223	410354	1.11	1.05	1.17	1.83E-04	0.00	3.17E-01
Lymphocytic Leukemia	74	Non-Hodgkin's Lymphoma	2411	410354	1.08	1.03	1.12	3.30E-04	0.00	5.48E-01
Melanoma	22	Breast	17901	219648	1.03	1.02	1.05	7.48E-05	0.63	9.95E-02
Melanoma	23	Oral Cavity / Pharynx	1223	410354	1.10	1.04	1.16	1.31E-03	0.50	1.56E-01
Oral Cavity / Pharynx	8	Lung	2488	410354	0.98	0.94	1.02	2.45E-01	0.00	7.55E-01
Oral Cavity / Pharynx	13	Melanoma	6782	410354	1.02	1.00	1.05	7.49E-02	0.24	2.52E-01
Oral Cavity / Pharynx	14	Non-Hodgkin's Lymphoma	2411	410354	1.10	1.06	1.15	2.66E-06	0.00	5.00E-01

Supplementary Figure 1. Odds ratios for significant associations between cancer-specific polygenic risk scores (PRS) and cancer outcomes, based on meta-analyses of European ancestry participants from the Genetic Epidemiology Research on Aging (GERA) cohort and UK Biobank. Odds ratios were estimated from logistic regression models, *p*-values were calculated from two-sided Wald tests, and statistical significance was determined according to a false discovery rate threshold of $q<0.05$. Cancers are ordered based on clustering of the odds ratios for each PRS across cancer outcomes.

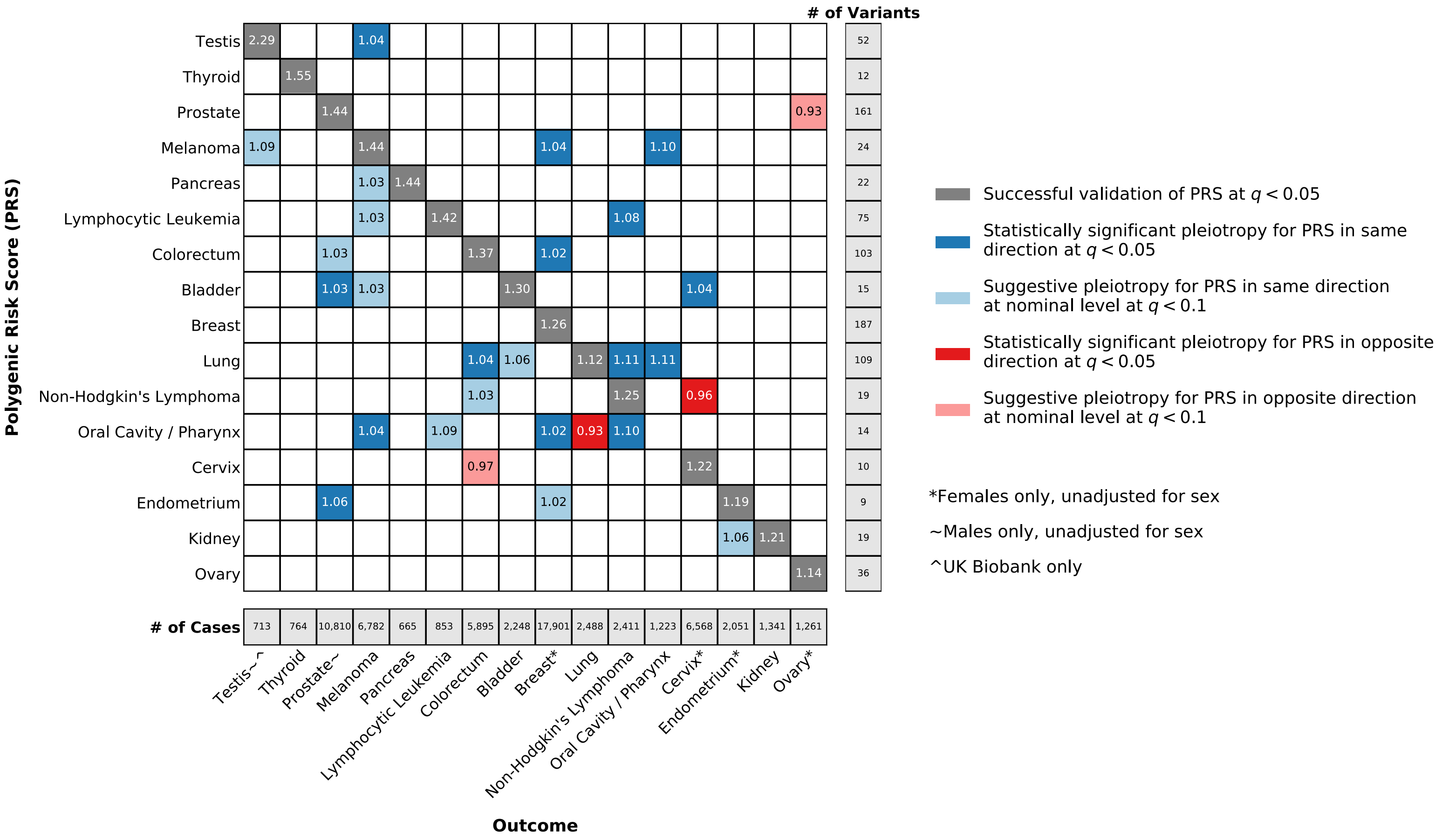

Supplementary Figure 2. Region enrichment for 141 significant novel and known pleiotropic risk variants compared to all PRS variants. The proportion of significant variants in each region is normalized by the region size relative to the genome size per kb.

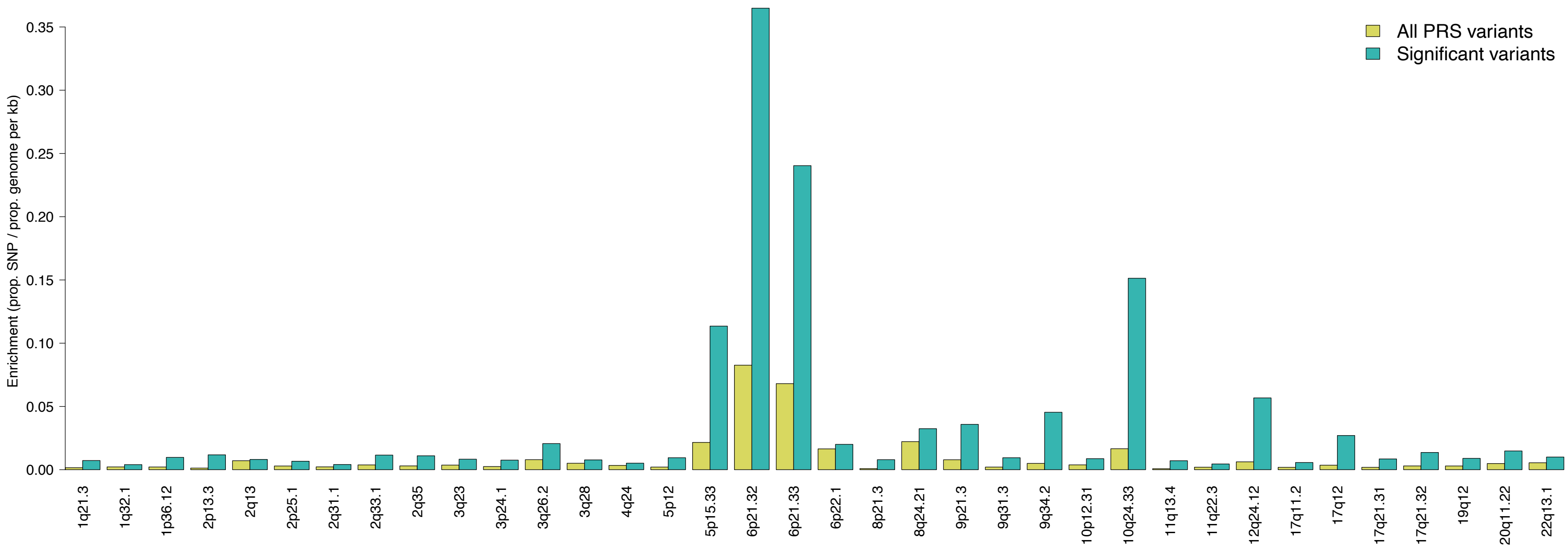

Supplementary Figure 3. Flow chart depicting the reduction of genome-wide significant associations abstracted from the literature to the variants included in the polygenic risk scores (PRS).

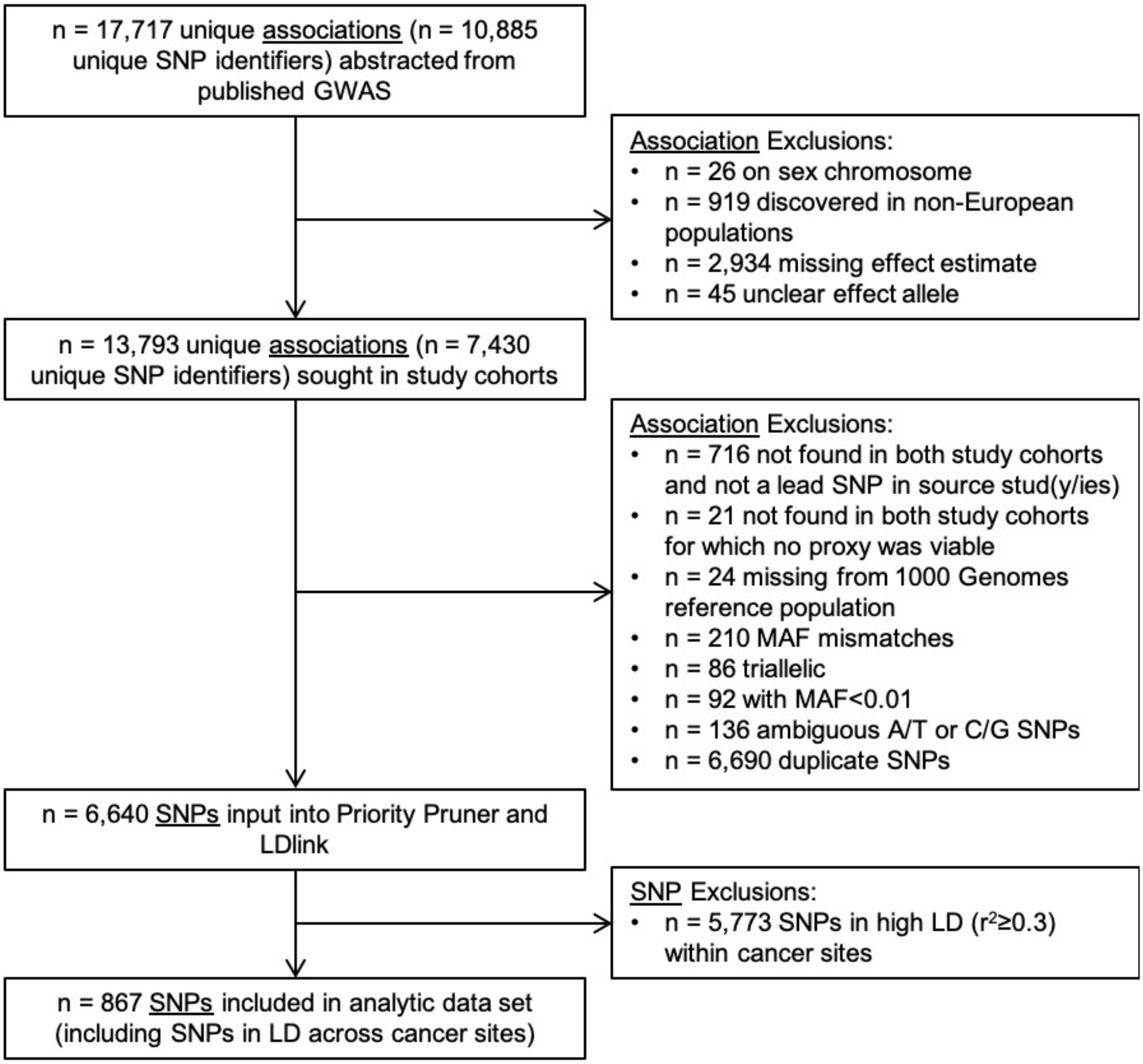

Supplement: Supplementary file 1 — Supplementary Information [file 41467_2021_21288_MOESM1_ESM.pdf]
