# Supplementary material for: Cross-cancer evaluation of polygenic risk scores for 16 cancer types in two large cohorts
Source: Nat Commun. 2021 Feb 12;12:970. doi: 10.1038/s41467-021-21288-z (PMC7880989; doi:10.1038/s41467-021-21288-z)
Supplement: Supplementary file 3 — Reporting Summary [file 41467_2021_21288_MOESM3_ESM.pdf]

nature research

Corresponding author(s): John S. Witte and Lori C. Sakoda

Last updated by author(s): Dec 24, 2020

Reporting Summary

Nature Research wishes to improve the reproducibility of the work that we publish. This form provides structure for consistency and transparency in reporting. For further information on Nature Research policies, see our Editorial Policies and the Editorial Policy Checklist.

Statistics

For all statistical analyses, confirm that the following items are present in the figure legend, table legend, main text, or Methods section.

n/a	Confirmed	
☐	☒	The exact sample size (n) for each experimental group/condition, given as a discrete number and unit of measurement
☐	☒	A statement on whether measurements were taken from distinct samples or whether the same sample was measured repeatedly
☐	☒	The statistical test(s) used AND whether they are one- or two-sided<br>*Only common tests should be described solely by name; describe more complex techniques in the Methods section.*
☐	☒	A description of all covariates tested
☐	☒	A description of any assumptions or corrections, such as tests of normality and adjustment for multiple comparisons
☐	☒	A full description of the statistical parameters including central tendency (e.g. means) or other basic estimates (e.g. regression coefficient) AND variation (e.g. standard deviation) or associated estimates of uncertainty (e.g. confidence intervals)
☐	☒	For null hypothesis testing, the test statistic (e.g. F, t, r) with confidence intervals, effect sizes, degrees of freedom and P value noted<br>*Give P values as exact values whenever suitable.*
☒	☐	For Bayesian analysis, information on the choice of priors and Markov chain Monte Carlo settings
☒	☐	For hierarchical and complex designs, identification of the appropriate level for tests and full reporting of outcomes
☐	☒	Estimates of effect sizes (e.g. Cohen's d, Pearson's r), indicating how they were calculated

Our web collection on statistics for biologists contains articles on many of the points above.

Software and code

Policy information about availability of computer code

Data collection	No software was used.
Data analysis	SAS v9.4 (https://support.sas.com/software/94/)<br>R v3.2.2 and v3.3.3 (https://cran.r-project.org)<br>PLINK v1.9 (https://www.cog-genomics.org/plink2)<br>KING v2.0 (http://people.virginia.edu/~wc9c/KING/)<br>QCTOOL v2 (https://www.well.ox.ac.uk/~gav/qctool_v2/)<br>VCFtools v0.1.13 (http://vcftools.sourceforge.net/)<br>PriorityPruner v0.1.4 (http://prioritypruner.sourceforge.net/)<br>LDLink v3.7 (https://ldlink.nci.nih.gov/?tab=home)<br>Python v3.5 (https://www.python.org/downloads/release/python-350/)<br>SHAPE-IT v2.5 (https://mathgen.stats.ox.ac.uk/genetics_software/shapeit/shapeit.html)<br>IMPUTE2 v2.3.1 (https://mathgen.stats.ox.ac.uk/impute/impute_v2.html)<br>Eigenstrat v4.2 (https://github.com/DReichLab/EIG/tree/master/EIGENSTRAT)<br>fastPCA part of EIGENSOFT package v6.0.1 (http://alimanfoo.github.io/2015/09/28/fast-pca.html)

For manuscripts utilizing custom algorithms or software that are central to the research but not yet described in published literature, software must be made available to editors and reviewers. We strongly encourage code deposition in a community repository (e.g. GitHub). See the Nature Research guidelines for submitting code & software for further information.

Data

Policy information about availability of data

All manuscripts must include a data availability statement. This statement should provide the following information, where applicable:

- Accession codes, unique identifiers, or web links for publicly available datasets
- A list of figures that have associated raw data
- A description of any restrictions on data availability

All results from this study are available from the article or Supplementary Materials. GERA data are available via application with a local collaborator at https://researchbank.kaiserpermanente.org/our-research/for-researchers/. UK Biobank data are publicly available from https://www.ukbiobank.ac.uk.

Field-specific reporting

Please select the one below that is the best fit for your research. If you are not sure, read the appropriate sections before making your selection.

☒ Life sciences ☐ Behavioural & social sciences ☐ Ecological, evolutionary & environmental sciences

For a reference copy of the document with all sections, see nature.com/documents/nr-reporting-summary-flat.pdf

Life sciences study design

All studies must disclose on these points even when the disclosure is negative.

Sample size

The Genetic Epidemiology Research on Adult Health and Aging (GERA) cohort is a prospective cohort of 102,979 adults drawn from >400,000 Kaiser Permanente Northern California (KPNC) health plan members who participated in the Research Program on Genes, Environment and Health. Following exclusions (described below), the GERA analytic population included 16,012 cases and 50,552 controls.

The UK Biobank is a population-based prospective cohort of 502,611 individuals from the United Kingdom. Following exclusions (described below), the UK Biobank analytic population included 48,969 cases and 359,802 controls.

The number of individuals included in this study was the maximum possible within GERA and the UK Biobank after exclusions made based on predetermined quality control criteria.

Data exclusions

Details about preliminary quality control procedures for GERA and the UK Biobank have been previously published. In addition, based on pre-specified exclusion criteria, we restricted to European ancestry individuals with matching self-reported and genetic sex. To further minimize population stratification, we excluded individuals for whom either of the first two ancestry PCs fell >5 standard deviations outside of the mean. We also removed samples with call rates <97%, heterozygosity >5 standard deviations from the mean, and/or first-degree relatives in the datasets.

Replication

In lieu of traditional replication, we successfully meta-analyzed our primary results across two independent samples. We note that the polygenic risk scores assessed were developed based on publicly available, previously published data. Our supplementary data file provides a full list of genetic variants used to construct each cancer-specific PRS, with the corresponding weights, to facilitate future replication of this work.

Randomization

Models were adjusted for age at specimen collection, first 10 ancestry PCs, sex (except models for sex-specific cancers), reagent kit used for genotyping (Axiom v1 or v2; GERA only), and genotyping array (UK Biobank only).

Blinding

As both GERA and the UK Biobank are population-based prospective cohort studies, there was no interventional component of our study. In addition, all data were de-identified prior to data analysis.

Reporting for specific materials, systems and methods

We require information from authors about some types of materials, experimental systems and methods used in many studies. Here, indicate whether each material, system or method listed is relevant to your study. If you are not sure if a list item applies to your research, read the appropriate section before selecting a response.

Materials & experimental systems

n/a	Involved in the study
☒	☐ Antibodies
☒	☐ Eukaryotic cell lines
☒	☐ Palaeontology and archaeology
☒	☐ Animals and other organisms
☐	☒ Human research participants
☒	☐ Clinical data
☒	☐ Dual use research of concern

Methods

n/a	Involved in the study
☒	☐ ChIP-seq
☒	☐ Flow cytometry
☒	☐ MRI-based neuroimaging

Human research participants

Policy information about studies involving human research participants

Population characteristics

GERA is a prospective cohort of 102,979 adults drawn from >400,000 Kaiser Permanente Northern California (KPNC) health plan members who participated in the Research Program on Genes, Environment and Health. Participants answered a baseline survey regarding lifestyle and medical history, provided a saliva specimen between 2008 and 2011, and were successfully genotyped.

The UK Biobank is a population-based prospective cohort of 502,611 individuals from the United Kingdom, ages 40 to 69 at recruitment between 2006 and 201058. Participants were evaluated at baseline visits during which assessment center staff introduced a touch-screen questionnaire, conducted a brief interview, gathered physical measurements, and collected biological samples.

Participants were more commonly female than male. GERA participants were older than UK Biobank participants (mean age: cases, 69 versus 60; controls, 62 versus 57).

Recruitment

GERA was developed from a mailed survey sent to all adult members of the Kaiser Permanente Medical Care Plan, Northern California Region (KPNC) who had been members for two years or more in 2007. The membership of KPNC is representative of the general population in the 14 county area in which facilities are located, although the membership is underrepresented for the extremes of income at both ends of the spectrum (dbGaP phs000674.v2.p2).

The UK Biobank is a population-based cohort of ~500,000 participants recruited in the United Kingdom between 2006-2010. Approximately 9.2 million individuals aged 40-69 years who lived within 25 miles of one of 22 assessment centers in England, Wales, and Scotland were invited to enter to cohort, and 5.5% participated in the baseline assessment. The UK Biobank is not representative of the general population on a variety of sociodemographic, physical, lifestyle and health-related characteristics, with evidence of a 'healthy volunteer' selection bias.

Ethics oversight

This study was approved by the KPNC and University of California Institutional Review Boards and the KP Research Bank and UK Biobank data access committees.

Note that full information on the approval of the study protocol must also be provided in the manuscript.
