# Supplementary material for: Cross-cancer evaluation of polygenic risk scores for 16 cancer types in two large cohorts
Source: Nat Commun. 2021 Feb 12;12:970. doi: 10.1038/s41467-021-21288-z (PMC7880989; doi:10.1038/s41467-021-21288-z)
Supplement: Supplementary file 4 — Description of Additional Supplementary Files [file 41467_2021_21288_MOESM4_ESM.pdf]

Description of Additional Supplementary Files

Supplementary Data 1. References for all GWAS from which we abstracted genome-wide significant risk variants

Supplementary Data 2. Variants included in the polygenic risk score for each cancer type

Supplementary Data 3. Detailed results from analyses of polygenic risk scores and cancer outcomes. Odds ratios were estimated from logistic regression models, p-values were calculated from two-sided Wald tests, and statistical significance ($p<0.05/16= 0.0031$) was determined accounting for multiple testing.

Supplementary Data 4. Polygenic risk score variants in linkage disequilibrium ($r2\geq0.3$) across cancer types

Supplementary Data 5. Associations between variants in all polygenic risk scores and each of the following cancer outcomes. Odds ratios were estimated from logistic regression models, p-values were calculated from two-sided Wald tests, and statistical significance ($p<0.05/798=6.3x10-5$) was determined correcting for the number of effective independent tests.

Supplementary Data 6. Significant cross-cancer variant associations. Odds ratios were estimated from logistic regression models, p-values were calculated from two-sided Wald tests, and statistical significance ($p<0.05/798=6.3x10-5$) was determined correcting for the number of effective independent tests.

Supplementary Data 7. Associations between cancer PRS and non-cancer phenotypes. Odds ratios (ever/never smoking status) were estimated from logistic regression models, and p-values were calculated from two-sided Wald tests. Risk ratios (remaining phenotypes) were estimated from linear regression models, and p-values were calculated from two-sided t-tests. Statistical significance ($p<0.05/20=0.0025$) was determined accounting for multiple testing.

Supplementary Data 8. Results from meta-analyses of polygenic risk scores and cancer outcomes restricted to incident and, separately, prevalent cases. Odds ratios were estimated from logistic regression models, and p-values were calculated from two-sided Wald tests.
