# Supplementary material for: Cross-cancer evaluation of polygenic risk scores for 16 cancer types in two large cohorts
Source: Nat Commun. 2021 Feb 12;12:970. doi: 10.1038/s41467-021-21288-z (PMC7880989; doi:10.1038/s41467-021-21288-z)
Supplement: Supplementary file 2 — Peer Review [file 41467_2021_21288_MOESM2_ESM.pdf]

Reviewer #1 (Remarks to the Author):

This is an impressive work by the investigators that evaluates pleiotropy of polygenetic risk scores (PRS) across 16 cancers and non-cancer traits among European ancestry using two cohorts (Genetic Epidemiology Research on Adult Health and Aging and the UK Biobank). The authors had a strong and rigorous study design The authors reported interesting associations of the cancer PRS with non-cancer phenotypes providing mechanistic insights of cancer risk.

However, the premise of evaluating PRS from one cancer with another cancer could be improved. The authors found that PRS from 11 cancers are associated with risk of another cancer, suggesting the genetic variants have pleiotropic effects and indirectly supporting prior studies of genetic correlation across cancers. But do these PRS findings actually help with understanding the biological mechanism of the disease? The PRS of another cancer may not be the best predictor of a cancer, i.e., not better than the cancer-specific PRS. What is really the next step with these results.

For some of the findings of cross cancer PRS associations, the PRS included variants that overlapped with risk of cancer. For example, the oral cavity/pharyngeal cancer showed bidirectional associations and they shared variants. Sensitivity analyses taking out the overlapping variants should be done to see if the relationship holds.

On page 7 and in the Discussion, the authors stated that they identified 60 novel SNP associations through their cross-cancer associations. However, they are using a much more relaxed threshold for statistical significance than what is used in specific cancer GWASs. I understand that the more relaxed threshold is because of fewer tests reported in this manuscript. However, most likely these SNPs were evaluated in the original GWAS but were declared not significant due to not meeting GWAS thresholds. Thus, one cannot claim them as novel risk variants for those particular cancers. Validation is certainly also needed.

The authors combined acute lymphoblastic leukemia (ALL) and chronic lymphocytic leukemia (CLL) together yet the etiology and clinical course are different. They should be separated. In fact, CLL is a subtype of NHL, another cancer considered herein. I agree that the NHL subtypes considered should be lumped together given the limited number of variants (under ten for each) identified for each NHL subtype (DLBCL, MZL, FL).

Why not include Myeloma, another blood cancer that have at least 23 GWAS variants that have been identified to date.

Page 19: why reduce the list of variants to those that were all independent of each other? Each PRS should have independent SNPs, but the reason to have independent SNPs across the PRS is not clear.

Reviewer #2 (Remarks to the Author):

This paper has developed polygenic risk scores developed for multiple types of cancer and subsequently considered if there were associated with other forms of cancer, tested within two large cohorts. They identified multiple, notable associations with the PRS and various other forms of cancer. Methods are appropriate and they have been deployed in two powerful cohorts. The paper is well written although the general message did feel a little disjointed at times.

Points
1. how do these results compare with other methods such as genetic correlations measured by LD score regression.
2 The sample sizes of the GWAS used to identify SNPs for the PRS differ markedly, how does this

influence the results?
3. have a number os sensitivity analysis been performed, for example SNPs with opposing effects (risk protective) across samples sizes have been described, are these results sensitive to this.
4. Exclusion inclusion of SNPs related to tobacco consumption?
5. Differences by cohort (UK and GERA)?
6. Differences histological subtype (breast lung) (as appropriate).
7. where either cohort included in the discovery phase of any of the GWAS?

Point-by-point responses to **Reviewer** comments on the manuscript
Cross-Cancer Evaluation of Polygenic Risk Scores for 16 Cancer Types in Two Large Cohorts

Reviewer #1 (Remarks to the Author):

This is an impressive work by the investigators that evaluates pleiotropy of polygenetic risk scores (PRS) across 16 cancers and non-cancer traits among European ancestry using two cohorts (Genetic Epidemiology Research on Adult Health and Aging and the UK Biobank). The authors had a strong and rigorous study design. The authors reported interesting associations of the cancer PRS with non-cancer phenotypes providing mechanistic insights of cancer risk.

Response: We thank the Reviewer for these positive comments and suggestions to strengthen our manuscript.

However, the premise of evaluating PRS from one cancer with another cancer could be improved. The authors found that PRS from 11 cancers are associated with risk of another cancer, suggesting the genetic variants have pleiotropic effects and indirectly supporting prior studies of genetic correlation across cancers. But do these PRS findings actually help with understanding the biological mechanism of the disease? The PRS of another cancer may not be the best predictor of a cancer, i.e., not better than the cancer-specific PRS. What is really the next step with these results.

Response: We agree with the reviewer that a PRS specific to a first cancer is not likely to better predict a second cancer than the PRS specific to the second cancer. We rather meant to suggest that the identification of cross-cancer PRS relationships might contribute to enhanced prediction. Given diagnosis with the first of two cancers with known shared genetic susceptibility, it could prove prudent to more aggressively consider primary prevention and screening efforts for the second cancer in the pair. Beyond prediction, biological insights into underlying etiologic mechanisms may be gained by investigating the functionality of shared variants. We have clarified these points in our updated Introduction (Page 4).

For some of the findings of cross cancer PRS associations, the PRS included variants that overlapped with risk of cancer. For example, the oral cavity/pharyngeal cancer showed bidirectional associations and they shared variants. Sensitivity analyses taking out the overlapping variants should be done to see if the relationship holds.

Response: Per the reviewer's suggestion, we conducted sensitivity analyses for the 11 statistically significant cross-cancer pairs by removing variants from the exposure PRS that are in linkage disequilibrium (LD; $r^2 > 0.3$) with variants known to be associated with the outcome cancer type. The variants we removed are described in Supplementary Table 6. The majority of the results were materially unchanged (see table below). Three relationships, however, were seemingly driven by variants in LD: endometrial cancer PRS and prostate cancer; oral cavity / pharyngeal cancer PRS and lung cancer; and oral cavity /

pharyngeal cancer PRS and melanoma. We describe these analyses in the manuscript on Pages 7 and 21.

PRS	Outcome	# PRS Variants (# Removed)	Primary Analysis	Sensitivity Analysis
Bladder	Cervix	15 (0)	$9.05x10^{-4}$	$9.05x10^{-4}$
Endometrium	Prostate	9 (1)	$5.33x10^{-9}$	**0.96**
Lung	Colorectum	109 (2)	$1.22x10^{-3}$	$1.27x10^{-3}$
Lung	Non-Hodgkin's Lymphoma	109 (5)	$5.57x10^{-7}$	$1.72x10^{-6}$
Lung	Oral Cavity / Pharynx	109 (6)	$1.06x10^{-4}$	$1.83x10^{-4}$
Lymphocytic Leukemia	Non-Hodgkin's Lymphoma	75 (1)	$1.49x10^{-4}$	$3.30x10^{-4}$
Melanoma	Breast	24 (2)	$6.34x10^{-7}$	$7.48x10^{-5}$
Melanoma	Oral Cavity / Pharynx	24 (1)	$7.84x10^{-4}$	$1.31x10^{-3}$
Oral Cavity / Pharynx	Lung	14 (6)	$6.24x10^{-4}$	**0.25**
Oral Cavity / Pharynx	Melanoma	14 (1)	$2.04x10^{-3}$	**0.075**
Oral Cavity / Pharynx	Non-Hodgkin's Lymphoma	14 (0)	$2.66x10^{-6}$	$2.66x10^{-6}$

On page 7 and in the Discussion, the authors stated that they identified 60 novel SNP associations through their cross-cancer associations. However, they are using a much more relaxed threshold for statistical significance than what is used in specific cancer GWASs. I understand that the more relaxed threshold is because of fewer tests reported in this manuscript. However, most likely these SNPs were evaluated in the original GWAS but were declared not significant due to not meeting GWAS thresholds. Thus, one cannot claim them as novel risk variants for those particular cancers. Validation is certainly also needed.

Response: We understand the Reviewer’s concern. We now describe the associations as “previously unreported” instead of “novel”.

The authors combined acute lymphoblastic leukemia (ALL) and chronic lymphocytic leukemia (CLL) together yet the etiology and clinical course are different. They should be separated. In fact, CLL is a subtype of NHL, another cancer considered herein. I agree that the NHL subtypes considered should be lumped together given the limited number of variants (under ten for each) identified for each NHL subtype (DLBCL, MZL, FL).

Response: The Reviewer makes an excellent point. We have thus conducted two series of sensitivity analyses: 1) evaluations of a CLL-specific PRS with respect to each cancer type and 2) evaluations of each PRS with respect to CLL. The manuscript now notes that the CLL-specific PRS was even more strongly associated with non-Hodgkin's lymphoma (OR=1.10; p=$1.97x10^{-6}$) than the non-specific lymphocytic leukemia PRS (OR=1.08; p=$1.48x10^{-4}$), which is consistent with CLL being a subtype of NHL (Pages 7-8). Results were otherwise materially unchanged.

Why not include Myeloma, another blood cancer that have at least 23 GWAS variants that have been identified to date.

Response: We restricted our analyses to the cancers with at least 650 cases across the GERA and UKB cohorts, which resulted in the exclusion of myeloma.

Page 19: why reduce the list of variants to those that were all independent of each other? Each PRS should have independent SNPs, but the reason to have independent SNPs across the PRS is not clear.

Response: We appreciate the opportunity to clarify our approach (Page 20). Each cancer-specific PRS was constructed by selecting independent variants (LD $r^2 < 0.30$). However, selected variants across PRS were not required to be independent.

Reviewer #2 (Remarks to the Author):

This paper has developed polygenic risk scores developed for multiple types of cancer and subsequently considered if there were associated with other forms of cancer, tested within two large cohorts. They identified multiple, notable associations with the PRS and various other forms of cancer. Methods are appropriate and they have been deployed in two powerful cohorts. The paper is well written although the general message did feel a little disjointed at times.

Response: We appreciate this positive feedback and hope that our revisions have improved the overall continuity and flow of key messages throughout our manuscript.

1. **How do these results compare with other methods such as genetic correlations measured by LD score regression?**

   Response: The updated Discussion now compares the results from our cross-cancer PRS analyses to those from analyses based on genome-wide assessments of pleiotropy. In doing so, we additionally note that PRS and genetic correlations capture distinct aspects of pleiotropy (Pages 12-13).

2. **The sample sizes of the GWAS used to identify SNPs for the PRS differ markedly, how does this influence the results?**

   Response: The larger the sample size in a GWAS, the larger the number of risk variants that are likely to be identified. While the extent of genetic contributions to a cancer also presumably impacts the number of genome-wide significant risk variants that have been discovered, cancers that have been evaluated in small GWAS generally have fewer known risk variants. As such, corresponding PRS will not be as well positioned to identify pleiotropy. We now address this point in the Discussion (Page 14).

3. **Have a number [of] sensitivity analysis been performed, for example SNPs with opposing effects (risk protective) across samples sizes have been described, are these results sensitive to this?**

Response: In response to comments from Reviewer #1, we have now conducted several sensitivity analyses. Those that removed variants from a PRS for one cancer in linkage disequilibrium with variants known to be associated with another cancer necessarily removed SNPs with opposing effects for the 11 statistically significant cross-cancer pairs. The following pairs included risk variants with r^2 > 0.3 and opposite risk alleles:

**Region**	**PRS 1**	**Variant 1**	**PRS 2**	**Variant 2**	**LD r^2**
5p15.33	Colorectum	rs2735940 (*TERT* upstream)	Lung	rs2853677 (*TERT* intronic)	0.62
6p21.3	Lung	rs115566240 (MHC intergenic)	Non-Hodgkin's Lymphoma	rs10484561 (MHC intergenic)	0.33
6p21.3	Lung	rs182364552 (MHC intergenic)	Non-Hodgkin's Lymphoma	rs115316013 (MHC intergenic)	0.31
5p15.33	Lung	rs380286 (*CLPTM1L* intronic)	Oral Cavity / Pharynx	rs467095 (*CLPTM1L* intronic)	0.96
5p15.33	Lung	rs62329683 (*TERT* upstream)	Oral Cavity / Pharynx	rs10462706 (*CLPTM1L* intronic)	0.66

Only the inverse association between the oral cavity pharyngeal cancer PRS and lung cancer became non-significant upon removing shared variants between the two cancers. We describe these findings in the manuscript on Pages 7 and 21.

4. **Exclusion inclusion of SNPs related to tobacco consumption?**

Response: Per the reviewer's suggestion, we have constructed alternative PRS that exclude variants demonstrating genome-wide significant associations with tobacco-related phenotypes in summary statistics from Liu, *et al.*, Nat Genet, 2019 Feb;51(2):237-244 (doi: 10.1038/s41588-018-0307-5). (For the 78 variants that were unavailable from these summary statistics, we evaluated associations in the UK Biobank directly. None demonstrated genome-wide significant associations.) The following variants were excluded from the noted PRS:

PRS	Chr	Pos	Variant	Current vs. Former	Ever vs. Never	Cigarettes / Day	Age Started
Breast	10	22032942	rs7072776		5.81E-11		
Lung	8	27344719	rs11780471		5.69E-12		7.00E-11
Lung	15	78782095	rs4887059			3.45E-89	
Lung	15	78825917	rs3885951			1.13E-35	
Lung	15	78857986	rs55781567	1.93E-08		1.03E-206	
Lung	15	78864024	rs56219480			3.83E-16	
Lung	15	78880752	rs2229961			1.17E-18	
Lung	15	78894759	rs3743078	1.34E-09		1.08E-118	
Lung	15	78906637	rs77438700			1.10E-48	
Lung	15	78909398	rs8192479			6.83E-10	
Lung	15	78953785	rs17487514			5.14E-56	
Lung	15	78953924	rs143527274			4.20E-09	

PRS	Chr	Pos	Variant	Current vs. Former	Ever vs. Never	Cigarettes / Day	Age Started
Lung	15	79018698	rs148832167			5.10E-29	
Lung	15	79042734	rs12905116			3.45E-22	
Lung	15	79043393	rs3861180			8.05E-10	
Lung	15	79074253	rs36061084			1.25E-60	
Lung	15	79105106	rs7182809			4.79E-19	
Lung	15	79110783	rs77719127			1.31E-29	
Lung	15	79132330	rs11072811			5.67E-33	
Lung	19	41337923	rs11879413	1.10E-09		2.85E-23	
Lung	19	41353107	rs56113850	2.52E-26		1.10E-81	
Prostate	2	63131731	rs721048				3.53E-10
Prostate	10	104414221	rs3850699		4.89E-08		

Even with tobacco-associated variants, the PRS for breast and prostate cancers were not initially implicated in any significant cross-cancer relationships. For lung cancer, however, we evaluated the updated PRS with respect to colorectal cancer, non-Hodgkin's lymphoma, and oral cavity / pharyngeal cancer. All three associations remained unchanged with the exclusion of tobacco-associated variants. The Results (Page 8) and Methods (Page 21) have been updated to describe these new analyses.

5. **Differences by cohort (UK and GERA)?**

   Response: Supplementary Tables 4b and 4c present results from the PRS analyses in GERA and the UK Biobank, respectively. In addition, Supplementary Table 4a describes any heterogeneity between the two cohorts. The Results also state that 1) none of the replicative within cancer PRS associations demonstrated significant heterogeneity ($p_{\text{Cochran's-Q}} < 0.05$) between the two cohorts (Page 6) and 2) the only significant cross-cancer PRS association that exhibited heterogeneity was that between the melanoma PRS and breast cancer association (I^2=0.79; $p_{\text{Cochran's-Q}}$=0.029, Page 7).

6. **Differences [in] histological subtype (breast lung) (as appropriate).**

   Response: We have now evaluated associations between each PRS and lung adenocarcinoma and squamous cell carcinoma (SCC) separately. We found that the oral cavity / pharyngeal cancer PRS was significantly inversely associated with lung adenocarcinoma (OR=0.89; p=1.13x10^{-4}) but not SCC (OR=0.94; p=0.20). We note, however, that there were fewer SCC (n = 488) than adenocarcinoma (n = 1006) cases. We describe these findings on Page 7. For cancer types other than lung cancer, one histology tends to dominate. Sample sizes were thus insufficient to consider heterogeneity across histology.

7. **[Were] either cohort included in the discovery phase of any of the GWAS?**

Response: As noted on Pages 14 and 19, we identified risk variants from one prior GWAS that included GERA and one prior GWAS that included the UK Biobank. The former studied prostate cancer and contributed effect estimates for only four out of 161 risk variants included in our prostate cancer PRS. The latter studied colorectal cancer, with the UK Biobank comprising only 9% of cases and 21% of the total sample size. We would expect any overfitting of the prostate and colorectal cancer PRS to be minimal given the limited overlap between our analytic data set and the source GWAS. In addition, because we are largely interested in cross-cancer associations, overfitting is not as pressing an issue.

Reviewer #1 (Remarks to the Author):

The authors responded to my prior comments

Reviewer #2 (Remarks to the Author):

I thank the reviewers for their consideration of my comments.

The authors have addressed most of my concerns, I still feel that there's a small clarification to be made regarding the distinction between the PRS approach and genetic correlations, why this is "unique" or "distinct". (pg 12-13) — this paragraph Is evidence presented that they are distinct? Is the lack of evidence in the PRS approach relative to the GC approach statistical power? That the PRS capture the strongest risk variants for a given phenotype, is also a function of statistical power, and this does vary by cancer type. Clarifying this to the reader would be useful.

I'd also suggest that genetic correlations capture the broader polygenic signal, which happens be to distributed across the genome, but "not across the genome" per-se.

Point-by-point responses to **Reviewer** comments on the manuscript
Cross-Cancer Evaluation of Polygenic Risk Scores for 16 Cancer Types in Two Large Cohorts

Reviewer #1 (Remarks to the Author):

The authors responded to my prior comments

> **Response:** We thank the Reviewer for reviewing our manuscript again and acknowledging that we have sufficiently addressed his or her comments.

Reviewer #2 (Remarks to the Author):

I thank the reviewers for their consideration of my comments.

> **Response:** We thank the Reviewer for reviewing our revised manuscript.

The authors have addressed most of my concerns, I still feel that there's a small clarification to be made regarding the distinction between the PRS approach and genetic correlations, why this is "unique" or "distinct". (pg 12-13) — this paragraph Is evidence presented that they are distinct? Is the lack of evidence in the PRS approach relative to the GC approach statistical power? That the PRS capture the strongest risk variants for a given phenotype, is also a function of statistical power, and this does vary by cancer type. Clarifying this to the reader would be useful.

I'd also suggest that genetic correlations capture the broader polygenic signal, which happens be to distributed across the genome, but "not across the genome" per-se.

> **Response:** We agree with the Reviewer that the PRS vs. genetic correlation approach required additional clarification. We have thus updated the noted paragraph (Pages 12-13) to incorporate the following content:
>
> *By capturing a broad polygenic signal, genetic correlations offer an overall sense of genetic sharing between two cancers. In contrast, PRS capture only the strongest risk variants for a given phenotype (which may in part be informed by the number of cases examined in contributing discovery GWAS). PRS thus enable more targeted analyses of cross-cancer susceptibility loci and have more clinical potential for individual risk assessment.*
